# Poster Session I - A92 NUTRITIONAL OPTIMIZATION TO REDUCE COMPLICATIONS FOLLOWING SURGERY FOR CROHN’S DISEASE: A QUALITY IMPROVEMENT PROJECT

**DOI:** 10.1093/jcag/gwaf042.092

**Published:** 2026-02-13

**Authors:** O Esenwa, B Bielawska

**Affiliations:** University of Ottawa, Ottawa, ON, Canada; Ottawa Hospital, Ottawa, ON, Canada

## Abstract

**Background:**

Most patients with Crohn’s disease (CD) who undergo CD surgery are at high risk of malnutrition and postoperative morbidity and mortality. Preoperative nutritional assessment should be the standard of care as it can significantly reduce complication rates and improve outcomes. However, patients undergoing elective surgery for CD at The Ottawa Hospital (TOH) often do not receive routine nutritional assessments. These patients are often malnourished, but this is not recognized until it is too late.

**Aims:**

1) Assess the current rates of formal nutrition assessment in patients undergoing elective CD surgery at TOH; 2) Determine what proportion of these patients develop post-operative complications.

**Methods:**

We performed retrospective chart reviews on patients who underwent elective or semi-elective CD surgery at TOH between June 2019 and October 2024. Patients were excluded if they had a diagnosis of ulcerative colitis or had a recent malignancy. Data collected included whether patients had a formal nutrition assessment and the timing in relation to their surgery. Outcome measures included 30-day post-operative complications (30-day infection, anastomotic leak or abscess formation, 90-day fistula development), 30-day follow-up surgery and 90-day readmission related to the surgery.

**Results:**

75 patients met inclusion criteria, 50.7% female with mean age 46. 70.7% of patients received a peri-operative nutrition assessment, with 41.3% assessed before surgery, 28% assessed at least 7 days before their surgery, and 29.3% only assessed after surgery. Of 42 patients who had malnutrition assessment documented, 88.1% had malnutrition and 35.7% were severely malnourished. 46.7% of patients had one or more of: post-operative complications, follow-up surgery or related re-admission. Of the patients who developed a complication, 29.4% had a nutrition assessment at least 7 days before surgery.

**Conclusions:**

Preoperative nutritional assessments and optimization can reduce the high risk of postoperative morbidity and mortality associated with elective CD surgery. Our study shows that current practices at TOH fall short of the expected standard of care. Despite a significant proportion of patients having evidence of malnutrition, less than half receive a nutrition assessment before surgery, and close to half develop post-operative complications. Our findings will help inform future intervention planning to standardize pre-surgical nutrition assessments for these patients.

**Funding Agencies:**

None

